# *N*-Ethyl-*N*-Nitrosourea (ENU) Mutagenesis Reveals an Intronic Residue Critical for *Caenorhabditis elegans* 3′ Splice Site Function *in Vivo*

**DOI:** 10.1534/g3.116.028662

**Published:** 2016-04-08

**Authors:** Omar A. Itani, Stephane Flibotte, Kathleen J. Dumas, Chunfang Guo, Thomas Blumenthal, Patrick J. Hu

**Affiliations:** * Institute of Gerontology and Departments of Internal Medicine and Cell and Developmental Biology, University of Michigan Medical School, Ann Arbor, Michigan 48109; † Department of Zoology, University of British Columbia, Vancouver, British Columbia V6T 123, Canada; ‡ Department of Molecular, Cellular, and Developmental Biology, University of Colorado, Boulder, Colorado 80309

**Keywords:** *C. elegans*, splicing, 3′ splice site, polypyrimidine tract, *daf-12*, ENU

## Abstract

Metazoan introns contain a polypyrimidine tract immediately upstream of the AG dinucleotide that defines the 3′ splice site. In the nematode *Caenorhabditis elegans*, 3′ splice sites are characterized by a highly conserved UUUUCAG/R octamer motif. While the conservation of pyrimidines in this motif is strongly suggestive of their importance in pre-mRNA splicing, *in vivo* evidence in support of this is lacking. In an *N*-ethyl-*N*-nitrosourea (ENU) mutagenesis screen in *Caenorhabditis elegans*, we have isolated a strain containing a point mutation in the octamer motif of a 3′ splice site in the *daf-12* gene. This mutation, a single base T-to-G transversion at the -5 position relative to the splice site, causes a strong *daf-12* loss-of-function phenotype by abrogating splicing. The resulting transcript is predicted to encode a truncated DAF-12 protein generated by translation into the retained intron, which contains an in-frame stop codon. Other than the perfectly conserved AG dinucleotide at the site of splicing, G at the –5 position of the octamer motif is the most uncommon base in *C. elegans* 3′ splice sites, occurring at closely paired sites where the better match to the splicing consensus is a few bases downstream. Our results highlight both the biological importance of the highly conserved –5 uridine residue in the *C. elegans* 3′ splice site octamer motif as well as the utility of using ENU as a mutagen to study the function of polypyrimidine tracts and other AU- or AT-rich motifs *in vivo*.

The mechanistic basis for pre-mRNA splicing is largely conserved in metazoans. Sequence motifs in pre-mRNA direct binding of spliceosomal components to 5′ and 3′ borders of introns and the subsequent assembly of a catalytically active spliceosome. The ensuing spliceosome-dependent 5′ and 3′ transesterification reactions result in intron excision and exon linkage (Morton and Blumenthal 2011).

*C. elegans* introns are defined by a 5′ splice site consensus that adheres to the canonical eukaryotic AG/GURAGU motif (Blumenthal and Steward 1997; Kent and Zahler 2000). Most metazoan 3′ splice sites contain a 10–12 nt polypyrimidine tract just upstream of the splice site. In mammals and other organisms, the polypyrimidine tract is recognized and bound by the large subunit of the 3′ splice site factor, U2AF, and the AG dinucleotide that defines the splice site itself is bound by the small subunit (Morton and Blumenthal 2011). In contrast, *C. elegans* 3′ splice sites are characterized by a highly conserved UUUUCAG/R octamer motif (Kent and Zahler 2000; Morton and Blumenthal 2011; Zahler 2012). Thus, while they are also pyrimidine-rich, *C. elegans* 3′ splice sites have evolved increased specificity compared to analogous sites in other metazoans. Nonetheless, the same recognition events occur as in other organisms; the large and small subunits of U2AF bind to the octamer motif (Hollins *et al.* 2005).

Although the entire UUUUCAG/R octamer is highly conserved, some nucleotides are almost invariant due to their key importance in U2AF recognition. The most conserved nucleotide is the –5 U (Kent and Zahler 2000). Furthermore, G is the least frequent base observed at this position (Kent and Zahler 2000), and replacement of the –5 U by G severely reduces U2AF binding (Hollins *et al.* 2005). These data are highly suggestive of an important role for the –5 U in splicing, and, indeed, it has been previously demonstrated that individual nucleotides in the octamer motif are crucial for splicing *in vivo* (Conrad *et al.* 1993; Zhang and Blumenthal 1996). However, until now, no mutation that perturbs splicing in a biologically relevant context by altering these nucleotides has been described.

Here we report the identification of a U-to-G transversion at the –5 position of the octamer motif of a 3′ splice site in *C. elegans* that abrogates splicing. The emergence of this mutant from a forward genetic screen provides an *in vivo* demonstration that a G at this position poisons 3′ splice site function.

## Materials and Methods

### C. elegans strains and maintenance

The following strains were used in this study: N2 Bristol, CB4856 (Wicks *et al.* 2001), RB759 *akt-1(ok525)* V (Hertweck *et al.* 2004), AA86 *daf-12(rh61rh411)* × (Antebi *et al.* 2000), and CB4037 *glp-1(e2141)* III (Priess *et al.* 1987). BQ29 *dpIr1* [N2 → CB4856, *eak-7(tm3188)*] IV; [N2 → CB4856, *akt-1(ok525)*] V) is a strain used for mapping in which *eak-7(tm3188)* and *akt-1(ok525)* were introgressed into the CB4856 background (Dumas *et al.* 2013a). The following mutant alleles were used: *eak-7(tm3188)* (Alam *et al.* 2010), *daf-9(dh6)* (Gerisch *et al.* 2001), and *smg-2(qd101)* (Richardson *et al.* 2011). A *daf-9(dh6)* mutant containing a rescuing extrachromosomal array encoding DAF-9::GFP (Gerisch *et al.* 2001) was provided by Adam Antebi. Double and triple mutants were constructed using standard techniques. Animals were maintained on nematode growth media (NGM) plates seeded with *Escherichia coli* OP50.

### ENU mutagenesis

*eak-7;akt-1* double mutant animals grown to the mid-L4 larval stage were incubated with 0.5 mM ENU in M9 buffer for 4 hr at room temperature with gentle agitation (De Stasio and Dorman 2001). Mutagenized animals were plated on NGM plates, allowed to recover overnight at 20°, and processed further as described (Dumas *et al.* 2013a).

### Isolation of dp664

Strain BQ9 was isolated from the suppressor of *eak-7;akt-1 (seak)* screen, and subjected to whole genome sequencing as previously described (Dumas *et al.* 2013a). Comparison of genome sequences from BQ9 and the parental nonmutagenized *eak-7(tm3188);akt-1(ok525)* double mutant strain revealed 39 nonsynonymous SNVs in BQ9. High resolution mapping using five X-linked nonsynonymous SNVs in BQ9 revealed that the causative mutation mapped near, and to the right of, the rightmost nonsynonymous SNV in the C07B5.4 gene. Examination of noncoding SNVs in this region revealed intronic SNVs in intron 2 of the *acr-8* gene (nucleotide 10,413,412, WormBase release WS238), and within the 3′ splice site of intron 13 of the *daf-12* gene (nucleotide 10,664,625, WS238), respectively. BQ9 was outcrossed with wild-type animals to isolate *dp664* from other X-linked SNVs. After six outcrosses, all X-linked nonsynonymous and intronic SNVs had been separated from *dp664*.

### Sequence analysis

Paired-end sequence reads were mapped to the *C. elegans* reference genome version WS230 (www.wormbase.org) using both short-read aligners BWA (Li and Durbin 2009) and Phaster (Philip Green, personal communication). The resulting alignment files were sorted and indexed, and SNVs were identified with the help of the SAMtools toolbox (Li *et al.* 2009).

### Dauer arrest assays

Dauer arrest assays were performed at 25° as previously described (Hu *et al.* 2006).

### Life span assays

Life span assays were performed at 20° as previously described (Dumas *et al.* 2013b).

### Reverse-transcriptase PCR

Total RNA was extracted from mixed-stage animals using TRIzol (Life Technologies) following the manufacturer’s recommendations. RNA was then reverse transcribed using the High Capacity cDNA Reverse Transcription Kit (Applied Biosystems) following the manufacturer’s recommendations. PCR primers (forward: 5′ CCGTATCAAGTTCCACCAGC 3′; reverse: 5′ GGATCAGAGCGGACAGAGAA 3′) were designed using Primer3 software (Untergasser *et al.* 2012) and purchased from Life Technologies. PCR was performed using Phusion High-Fidelity DNA polymerase (New England BioLabs) in a Mastercycler ep *realplex* thermal cycler (Eppendorf North America). PCR products were subjected to 1.5% agarose gel electrophoresis.

### Data availability

All data and reagents are freely available upon request. The authors state that all data necessary for confirming the conclusions presented in the article are represented fully within the article.

## Results and Discussion

**Figure 1:**
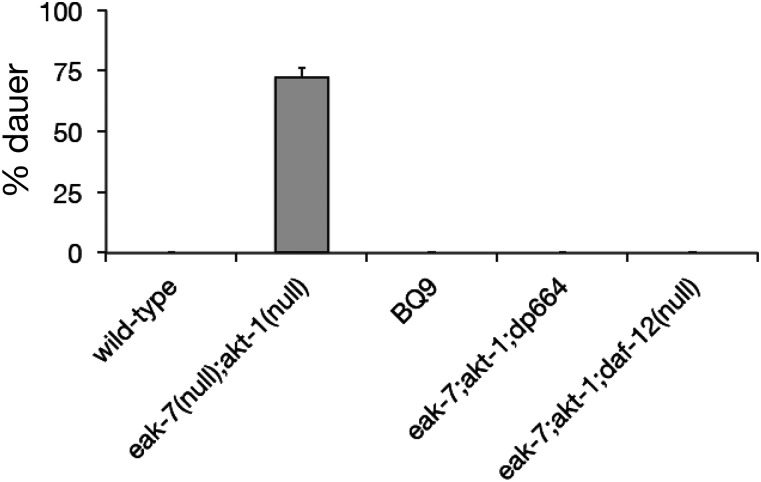
The *dp664* mutation suppresses the dauer-constitutive phenotype of *eak-7;akt-1* double mutants. BQ9 is the mutant strain that emerged from the *eak-7;akt-1* suppressor screen, and *eak-7;akt-1;daf-12(dp664)* is the triple mutant constructed after *daf-12(dp664)* had been outcrossed six times with wild-type animals to remove other mutagen-induced SNVs in the BQ9 strain. Notably, the outcrossed strain containing *daf-12(dp664)* was devoid of all mutagen-induced nonsynonymous SNVs present on the X chromosome in BQ9.

**Figure 2:**
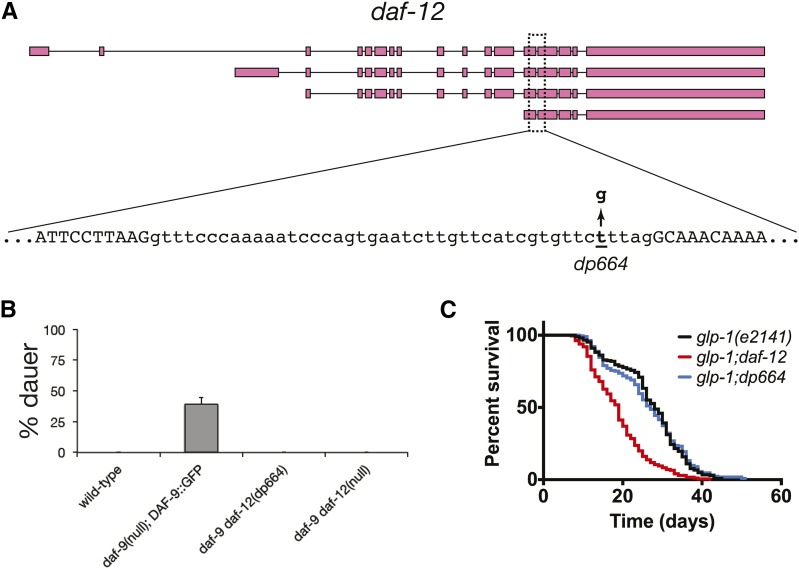
The *daf-12(dp664)* mutation reduces *daf-12* activity. (A) Location of the *daf-12(dp664)* SNV in the *daf-12* genomic locus. (B) *daf-12(dp664)* fully suppresses the dauer-constitutive phenotype of a *daf-9* null mutation. Results shown are the composite of two independent experiments. (C) *daf-12(dp664)* does not suppress life span extension in animals lacking a germline. Results shown are the composite of three independent experiments.

We sought to identify new regulators of *C. elegans* dauer arrest by performing an ENU-based forward genetic screen for suppressors of the *eak-7;akt-1* dauer-constitutive (*seak*) phenotype (Figure 1; see *Materials and Methods* for details) (Dumas *et al.* 2013a; Itani *et al.* 2015). Single nucleotide polymorphism mapping (Davis *et al.* 2005) of one mutant strain, BQ9, established that the *seak* mutation in BQ9 was X-linked and also excluded as causative mutations all five nonsynonymous X-linked single nucleotide variants (SNVs) that had been identified by whole genome sequencing. Further mapping established tight linkage of the noncoding *dp664* SNV with the *seak* phenotype. The *dp664* SNV lies within the 3′ splice site octamer motif of intron 13 of the *daf-12* gene (Figure 2A), which encodes a nuclear receptor that is required for dauer arrest and germline ablation-induced longevity (Antebi *et al.* 1998, 2000; Hsin and Kenyon 1999).

As *daf-12* loss-of-function is known to suppress the dauer-constitutive phenotype of *eak-7;akt-1* double mutants (Alam *et al.* 2010), as well as many other dauer-constitutive mutants (Riddle *et al.* 1981; Vowels and Thomas 1992), we tested the possibility that *daf-12(dp664)* was responsible for the *seak* phenotype. *daf-12(dp664)* was separated from all nonsynonymous X-linked SNVs, and all but one noncoding SNV, by outcrossing (see *Materials and Methods* for details). Construction of an *eak-7;akt-1;daf-12(dp664)* triple mutant revealed that *daf-12(dp664)* suppressed *eak-7;akt-1* dauer arrest as strongly as the *daf-12* null allele *rh61rh411* (Figure 1).

To establish that *daf-12(dp664)* causes a loss of *daf-12* function, we tested *daf-12(dp664)* for its ability to suppress the dauer-constitutive phenotype of the *daf-9* null allele *dh6* (Gerisch *et al.* 2001). *daf-9* encodes a 3-keto-sterol-26-monooxygenase that catalyzes the last step in the biosynthesis of dafachronic acids (DAs), which are steroid ligands for DAF-12 (Motola *et al.* 2006). In the absence of *daf-9* activity, unliganded DAF-12 induces nonconditional dauer arrest; in animals with wild-type *daf-9* activity, DAs promote reproductive development by binding to DAF-12 (Motola *et al.* 2006).

*daf-9(dh6)* mutants arrest nonconditionally as partial dauers, and this phenotype is fully suppressed by a *daf-12* null mutation (Figure 2B) (Gerisch *et al.* 2001). Because this nonconditional dauer-constitutive phenotype precluded propagation of *daf-9(dh6)* animals, we used a transgenic *daf-9(dh6)* strain harboring a rescuing extrachromosomal DAF-9::GFP transgene (Gerisch *et al.* 2001) as a positive control for dauer arrest (Figure 2B). Progeny of *daf-9*; Ex[DAF-9::GFP] transgenic animals were scored for dauer arrest. As expected, all non-dauer animals expressed GFP, whereas no dauer progeny had visible GFP. *daf-12(dp664)* suppressed *daf-9* dauer arrest to the same extent as *daf-12(null)* (Figure 2B). Since *daf-12* mutations are the only known dauer-defective mutations that suppress the dauer-constitutive phenotype of *daf-9* loss-of-function mutations (Gerisch *et al.* 2001), this result strongly suggests that the *daf-12(dp664)* mutation causes a loss of *daf-12* activity.

As *daf-12* is also required for life span extension in animals lacking a germline (Hsin and Kenyon 1999), we determined the influence of *daf-12(dp664)* on the life spans of germline-ablated animals. We assayed *glp-1(e2141)* mutant animals, which harbor a temperature-sensitive *glp-1* mutation that prevents germline development when animals are raised at the restrictive temperature (Priess *et al.* 1987; Arantes-Oliveira *et al.* 2002). In contrast to *daf-12(null)*, which shortened the life span of germline-ablated *glp-1* mutant animals as expected (Hsin and Kenyon 1999; Dumas *et al.* 2013b), the *daf-12(dp664)* mutation did not significantly influence the life span of animals lacking a germline (Figure 2C). Taken together with our molecular analysis of *daf-12(dp664)* (see below), this result suggests that *daf-12(dp664)* may retain DAF-12 activities that promote longevity in the absence of a germline.

**Figure 3:**
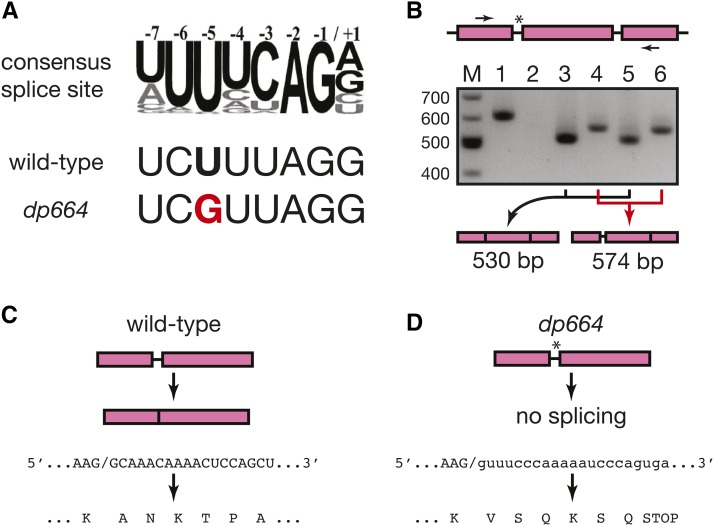
The *daf-12(dp664)* mutation abrogates splicing of intron 13 of the *daf-12* gene. (A) Alignment of the consensus 3′ splice site motif (Kent and Zahler 2000; Hollins *et al.* 2005) with the intron 13 3′ splice site sequence of *daf-12* in wild-type and *daf-12(dp664)*. (B) RT-PCR of *daf-12* cDNA spanning the *daf-12(dp664)* mutation. Top: schematic of exons 13-15 and introns 13-14 of the *daf-12* gene. Arrows denote primers used for PCR amplification. The asterisk denotes the location of the *daf-12(dp664)* mutation. Middle: 1.5% agarose gel electrophoresis of PCR products using cDNA templates from various strains. Lanes: M: molecular weight markers; 1: genomic DNA template control; 2: wild-type cDNA template control with reverse transcriptase omitted; 3: wild-type; 4: *daf-12(dp664)*; 5: *smg-2(qd101)*; 6: *smg-2;daf-12(dp664)*. Bottom: structure of the predominant PCR product in lanes 3-6, deduced from Sanger sequencing of excised and purified DNA fragments. (C) Deduced amino acid sequence of DAF-12 translated from wild-type cDNA from the exon 13/14 splice junction. (D) Deduced amino acid sequence of DAF-12 translated from *daf-12(dp664)* cDNA.

To determine the molecular basis for loss of *daf-12* function in *daf-12(dp664)*, we analyzed splicing of *daf-12* transcripts in wild-type and *daf-12(dp664)* animals. The *daf-12(dp664)* mutation is a T-to-G transversion that results in a U-to-G mutation in the 3′ splice site octamer motif of the 13th intron of *daf-12* pre-mRNA (Figure 2A) (Antebi *et al.* 2000). Other than the invariant AG dinucleotide at positions –2 and –1, this uridine at position –5 is the most highly conserved residue in *C. elegans* 3′ splice sites (Figure 3A) (Kent and Zahler 2000).

Oligoribonucleotides containing the UUUUCAG/G 3′ splice site consensus bind to the U2AF splicing factor *in vitro* (Zorio and Blumenthal 1999), and mutation of the –5 U to G strongly reduces U2AF binding (Hollins *et al.* 2005), suggesting that this mutation could potentially abrogate the excision of this intron and/or reveal alternative splicing at a cryptic downstream 3′ splice site. We tested these possibilities by amplifying DNA fragments encompassing the 13th intron, 14th exon, and 14th intron of *daf-12* after reverse transcription of total RNA isolated from wild-type and *daf-12(dp664)* animals (Figure 3B). We also tested RNA templates from animals containing a *smg-2* nonsense mutation (Richardson *et al.* 2011) to facilitate the detection of abnormally spliced transcripts that might be metabolized through nonsense-mediated degradation (Zhang and Blumenthal 1996). PCR amplified a DNA fragment with electrophoretic mobility corresponding to the expected 631 bp fragment from a genomic DNA template (Figure 3B, lane 1). No bands were visible in a control sample using RNA template processed without reverse transcriptase (lane 2), indicating that any potential DNA contamination of the total RNA preparation was present in quantities below the limit of detection by PCR amplification in this assay. A DNA fragment with greater electrophoretic mobility than the genomic product was amplified from reverse-transcribed RNA from wild-type (lane 3) and *smg-2* mutant animals (lane 5). Sanger sequencing of these gel-purified products confirmed their identity as normally spliced *daf-12* cDNAs of the predicted size of 530 bp (Figure 3, B and C) (Antebi *et al.* 2000). *smg-2* mutation did not result in the amplification of additional products not present in the wild-type sample (cf. lanes 3 and 5), suggesting that this region of the *daf-12* pre-mRNA is not subject to alternative splicing.

A DNA fragment with electrophoretic mobility greater than fragments generated from a genomic DNA template, and distinct from fragments produced from reverse-transcribed wild-type RNA was amplified from RNA isolated from both *daf-12(dp664)* (lane 4) and *smg-2;daf-12(dp664)* (lane 6). Sequencing of gel-purified products revealed that these products are cDNAs in which the 13th intron is retained (Figure 3, B and D). The structure of these cDNAs conforms exactly to the cDNA structure predicted if the T-to-G transversion abrogates splicing of the 13th intron. Translation into the retained intron results in premature termination due to the presence of an in-frame termination codon (Figure 3D). This is predicted to create a truncated DAF-12 protein that contains a DNA binding domain but lacks a C-terminal ligand-binding domain (Antebi *et al.* 2000). As this transcript is detectable and not significantly affected by *smg-2* mutation (Figure 3B, compare lanes 4 and 6), this mutant DAF-12 protein may promote longevity in the context of germline ablation (Figure 2C), even though it does not suffice to induce dauer arrest in the absence of *daf-9* activity (Figure 2B). As is the case in the wild-type *daf-12* background, *smg-2* mutation did not reveal new amplified products that were not detectable in animals with intact nonsense-mediated decay (Figure 3B, compare lanes 4 and 6). Therefore, *daf-12(dp664)* does not expose downstream cryptic 3′ splice sites in *daf-12* pre-mRNA.

To our knowledge, this is the first polypyrimidine tract mutation that has been shown to reduce gene activity in a physiologic context by abrogating 3′ splice site function. Our discovery of a single base mutation that abrogates splicing highlights the importance of the –5 uridine residue to 3′ splice site function, and is consistent with the observation that, other than the AG dinucleotide that defines the 3′ splice junction, the –5 uridine is the most highly conserved residue in the 3′ splice site octamer motif (Kent and Zahler 2000). Features of this specific splice site likely facilitated the isolation of this mutation. The wild-type sequence of this octamer motif, UCUUUAG/G, deviates from the consensus motif, UUUUCAG/G, at positions –6 and –3 relative to the splice site (Figure 3A). U-to-C mutation at the –6 position and C-to-U mutation at the –3 position both reduce binding of U2AF to the octamer motif (Hollins *et al.* 2005). Therefore, this particular wild-type 3′ splice site may be more dependent upon the uridine residue at the –5 position for proper splicing than other splice sites that adhere more closely to the consensus motif. Furthermore, although mutation of the –5 uridine to any residue reduces binding to U2AF, a U-to-G mutation reduces binding to a greater extent than U-to-A or U-to-C mutations (Hollins *et al.* 2005).

**Figure 4:**
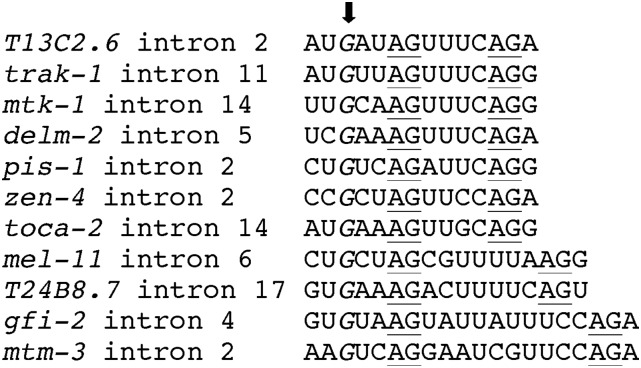
Examples of paired 3′ splice sites with a G at –5 in the octamer motif of the upstream site. AG dinucleotides used for splicing are underlined. All examples are supported by evidence from expressed sequence tags and/or whole transcriptome profiling reads. Sequences are aligned at the upstream splice sites, and the G residues at the –5 position are italicized and denoted by the arrow. Note the divergence of the upstream octamer motifs from the UUUUCAG/R consensus.

Genome-wide analysis of *C. elegans* 3′ splice sites indicates that G is the most uncommon nucleotide present at the conserved –5 position of the UUUUCAG/R octamer motif, occurring at 0.3% of canonical *C. elegans* 3′ splice sites (Kent and Zahler 2000). Nonetheless, there exist several examples of splicing events at 3′ splice sites with a G at the –5 position (Figure 4). Interestingly, they appear to represent instances of paired splice sites, separated by 6, 9, or 12 bp. In each case the site containing the G at –5 is the more 5′ site, and the site has an overall poor match to the octamer motif (Figure 4). The bias in favor of spacing in multiples of 3 bp may be due to the need for both splice sites to produce an in-frame mRNA. Presumably, there are other such cases with different spacing where the mRNA resulting from splicing at the upstream position is subject to nonsense-mediated decay (Zhang and Blumenthal 1996).

These kinds of paired splice sites in nematodes have been extensively analyzed by Ragle *et al.* (2015). This group showed that the upstream site, which lacks the binding site for the 65 kDa subunit of U2AF, is used principally in the germline (Ragle *et al.* 2015). A reasonable idea for how these paired splice sites arise is that the downstream site with a U at –5 binds U2AF to locate the 3′ splice site in the vicinity, causing branch formation a short distance upstream [in *C. elegans*, there is no branchpoint consensus sequence (Blumenthal and Steward 1997)]. Then the second step of splicing can occur at any AG dinucleotide that occurs a short distance downstream of the branchpoint. In this model, the splice site that is recognized by U2AF locates the branchpoint, but U2AF recognition is not needed for the actual splicing event. Hence, a G is tolerated at these upstream splice sites because it resides within a motif that is distinct from the one bound by U2AF. The idea that the primary or sole function of U2AF is to correctly position a branchpoint for accurate splicing may be specific for instances like *C. elegans* where there is no direct branchpoint recognition prior to the U2 snRNP interaction. In the case of the *daf-12(dp664)* mutation, there is no good match to the octamer consensus just downstream, so the splice site is inactivated by the –5 U-to-G mutation.

The prevalent use of ethyl methanesulfonate (EMS) as a mutagen in forward genetic screens in *C. elegans* has likely biased such screens against the isolation of loss-of-function splicing mutations in the octamer motif, since the mutational bias of EMS in favor of G-to-A and C-to-T transitions (Flibotte *et al.* 2010) would reduce the frequency of point mutations affecting the conserved uridines. In this regard, the use of ENU as a mutagen in genetic screens may reveal more information about octamer motif function *in vivo*, as ENU has less mutational bias than EMS (Flibotte *et al.* 2010). This feature of ENU suggests that it may also be particularly useful in identifying biologically important features of AU- or AT-rich motifs that govern nucleic acid function and/or regulation.
